# Dual-Responsive Polypropylene Meshes Actuating as Thermal and SERS Sensors

**DOI:** 10.1021/acsbiomaterials.2c00334

**Published:** 2022-06-02

**Authors:** Sonia Lanzalaco, Pau Gil, Júlia Mingot, Alba Àgueda, Carlos Alemán, Elaine Armelin

**Affiliations:** † Departament d’Enginyeria Química, IMEM-BRT, EEBE, Universitat Politècnica de Catalunya, C/Eduard Maristany, 10-14, Ed. I, Second Floor, 08019, Barcelona, Spain; ‡ Barcelona Research Center in Multiscale Science and Engineering, Universitat Politècnica de Catalunya, C/Eduard Maristany, 10-14, Basement S-1, 08019, Barcelona, Spain; § Departament d’Enginyeria Química, CERTEC, EEBE, Universitat Politècnica de Catalunya, C/Eduard Maristany, 10-14, Ed. I, Fifth floor, 08019, Barcelona, Spain; ∥ Institute for Bioengineering of Catalonia (IBEC), The Barcelona Institute of Science and Technology, Baldiri Reixac 10-12, 08028, Barcelona, Spain

**Keywords:** polypropylene, poly(*N*-isopropylacrylamide), surface functionalization, gold nanoparticles, SERS spectroscopy

## Abstract

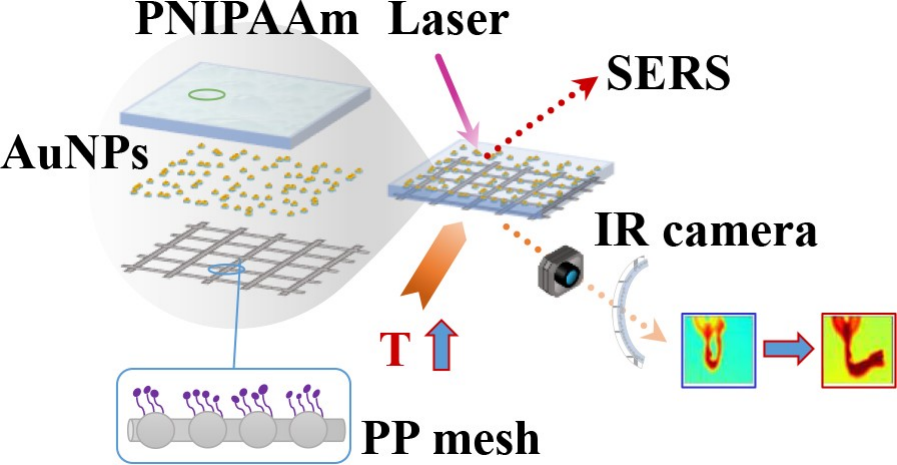

Polypropylene (PP) surgical meshes, with different knitted architectures, were chemically functionalized with gold nanoparticles (AuNPs) and 4-mercaptothiazole (4-MB) to transform their fibers into a surface enhanced Raman scattering (SERS) detectable plastic material. The application of a thin layer of poly[*N*-isopropylacrylamide-*co*-*N,N′*-methylene bis(acrylamide)] (PNIPAAm-*co*-MBA) graft copolymer, covalently polymerized to the mesh-gold substrate, caused the conversion of the inert plastic into a thermoresponsive material, resulting in the first PP implantable mesh with both SERS and temperature stimulus responses. AuNPs were homogeneously distributed over the PP yarns, offering a clear SERS recognition together with higher PNIPAAm lower critical solution temperature (LCST ∼ 37 °C) than without the metallic particles (LCST ∼ 32 °C). An infrared thermographic camera was used to observe the polymer-hydrogel folding-unfolding process and to identify the new value of the LCST, connected with the heat generation by plasmonic-resonance gold NPs. The development of SERS PP prosthesis will be relevant for the bioimaging and biomarker detection of the implant by using the plasmonic effect and Raman vibrational spectroscopy for minimally invasive interventions (such as laparoscopy), to prevent patient inflammatory processes. Furthermore, Raman sources have been proved to not damage the cells, like happens with near-infrared irradiation, representing another advantage of moving to SERS approaches. The findings reported here offer unprecedented application possibilities in the biomedical field by extrapolating the material functionalization to other nonabsorbable polymer made devices (e.g., surgical sutures, grapes, wound dressings, among others).

## 1 Introduction

Recent advances in polymer chemistry serve as the foundations for rapid progress in novel classes of sensor technologies for medical applications.^1−7^ The combination of polymers and metal coatings (and/or polymer@metal particles), together with the micro- and macro-fabrication of electrodes, provide broad capabilities in continuous biophysical and biochemical measurements of health status, in many cases with levels of precision and accuracy that can compete with clinical detection standards.^8^ One example is the recent work published by Seo et al.,^9^ where they developed a gold nanonetwork (Au NN)-based microelectrode adaptable to monitor neural activities (called “electrocorticogram”, ECoG). The system was tested as an implantable neural electronic in mice brains. The *ex vivo* brain activity was monitored with electrochemical impedance spectroscopy (EIS) and by ultraviolet–visible (UV–vis) spectroscopy after Au-NN light stimulation. They also performed *in vivo* tests for mapping of ECoG neural signals to fully decode a neural circuit, after light stimulation and without any photoelectric external artifact. In another example, Gracias and co-workers fabricated a “smart” medical device for applications as a 3D biosensing platform.^10^ They prepared an ultrathin and flexible skin that is capable of conformably wrapping soft or irregularly shaped 3D biological cancer cells and pollen grain. The detection of such structures was made with 3D label-free spatially resolved molecular spectroscopy via surface-enhanced Raman scattering (SERS). Their platform consisted of an ultrathin thermally responsive PNIPAAm-graphene-nanoparticle hybrid skin that can self-fold and wrap around 3D micro-objects in a conformal manner. Therefore, the use of thermosensitive hydrogel (TSH)^11^ together with other materials drives forces to the employment of such patterned devices, in combination with powerful spectroscopy tools (SERS, SEIRAS, UV–vis-NIR), for materials’ sensing detection. There are several other examples of Au functionalization or deposition in different materials.^12,13^ However, most of them are based on the utilization of planar and flat surfaces, which have usually fewer detection complications than samples with complex architectures, such as those used in biomedicine (wounds, sutures, stent, meshes, catheters, among others).

Hong and co-workers^14^ have recently highlighted the importance of using flexible SERS substrates for either engineering applications or biomedical in situ diagnostics, as promising materials for the next generation of wearable sensors in the near future. For instance, SERS spectroscopy has demonstrated important advantages in biosensor applications because of its high sensitivity and multiplexing ability (detection and bioimaging tools).^15^ Moreover, the chemical composition of the nanometallic particles is versatile and can be varied by combining magnetic particles and conducting materials to modulate the target applications.^16,17^ Thus, SERS technology has enabled great advancements toward *in vivo* applications.

The pioneering study that described a method for the covalent deposition of AuNPs in polypropylene mesh fibers was published by Quidant and co-workers in 2016.^18,19^ The authors were able to kill a resistant bacteria strain (*Staphylococcus aureus*) by using homogeneous citrate-stabilized gold nanorods (GNRs) immobilized on the plastic surface and by further applying local light irradiation with near-infrared spectroscopy (NIR), thus creating the first surgical implant able to self-destruct biofilm growth. The mechanism is based on the localized heating of the surgical mesh by infrared light. Although nowadays NIR light irradiation is being used in photothermal therapy (PTT)^20,21^ as an emerging technology that combines the material surface modification with metal nanomaterials (nanorods, nanoparticles, nanocubes, nanostars, etc.) in bioimaging, bacterial inhibition, and anticancer applications, the control of the light intensity in biological tissues is a great issue because an overheating of live cells can damage them irreversibly.

Recent advances in the employment of plasmonic NPs serve as the foundations for rapid progress in unusual spectroscopy applications of SERS in medical field.^22,23^ Although recent and renowned research is behind the development of this type of bioimaging and biomarker detection by using plasmonic effect and Raman vibrational spectroscopy,^15,24^ to the best of our knowledge, Roth & Theato^25^ were the first in reporting the graft reaction between AuNPs and stimuli responsive hydrogels in 2008. After such pioneering work, in 2009, Álvarez-Puebla & Liz-Marzán reported the effectiveness of AuNPs with PNIPAAm as shell (Au@pNIPAM) for SERS tag applications.^26^ More recently, Bodelón et al.^27^ were able to detect three tumor-associated protein biomarkers (*in vitro*) by using Au@pNIPAM microgels and an excitation laser of 633 nm. Both works proved the potential applicability of such particles in combination with thermoresponsive hydrogels for biomedical diagnosis.

The present work, which reports the first example of a plasmon-enabled thermosensitive PP mesh, was motivated by the need of finding sensors for imaging diagnosis and noninvasive (or minimally invasive) detection of the implant by using spectroscopy tools. For this purpose, a yarn’s surface has been functionalized with spherical AuNPs, trapping 4-mercaptobenzonitrile (4-MB) molecules as Raman reporter (RaR). Then, a thin layer of PNIPAAm, copolymerized with *N*,*N*′-methylene bis(acrylamide) as cross-linker, has been deposited on the PP fibers and monitored by SERS and an infrared camera. Therefore, the most important challenge of this work, with respect to previous studies, is the advantage of combining SERS *in situ* analysis and the conductivity properties of the Au metallic particles to potentiate the folding-unfolding behavior of PNIPAAm hydrogel, leading to both diagnosis and thermal successful sensor responses.

## 2 Experimental Procedure

### 2.1 Materials

Two types of meshes used for hernia repair, manufactured with isotactic polypropylene yarns, were used as substrates for grafting reactions: (i) with low- (PP-LD) and (ii) mid-(PP-MD) surface density (36 g/m^2^ and 48 g/m^2^, respectively). The pore sizes were 1.0 mm^2^ and 2.8–3.6 mm^2^ for PP-LD and PP-MD, respectively. Samples were kindly supplied by B. Braun Surgical S.L.U. Tetrachloroauric(III) acid trihydrate (HAuCl_4_·3H_2_O, Reagent Plus >99%, CAS 16961-25-4); sodium citrate dehydrate (Na_3_C_6_H_5_O_7_·2H_2_O, Reagent Plus >99%, CAS 6132-04-3); 4-mercaptobenzonitrile (4-MB, Reagent Plus >99%, CAS 36801-01-1); ethylenediamine (ED) (ReagentPlus ≥99%, CAS 107-15-3); *N*-isopropylacrylamide (NIPAAm) monomer (purity 99%, CAS 2210-25-5), *N*,*N*′-methylene bis(acrylamide) (MBA) cross-linker (Reagent Plus 99%, CAS 110-26-9); and *N*,*N*,*N*′,*N*′-tetramethylethylenediamine (TEMED, Reagent Plus 99%, CAS110-18-9) initiator, were supplied by Sigma-Aldrich (Spain). Ammonium persulfate catalyst (APS, purity 98% CAS7727-54-0) was provided by Panreac S.A. All reagents were used as received. Milli-Q water grade (0.055 S cm^–1^) was used in all synthetic processes. Nitrogen gas was used for the radical polymerization reactions and was of pure grade (99.995% purity).

### 2.2 Synthesis and Characterization of Gold Nanoparticles (AuNPs)

For the synthesis of AuNPs with suitable size, a method developed by Bastús et al. was used.^28^ To produce the gold seeds, 150 mL of sodium citrate solution (2.2 mM) were heated to 100 °C in a 200 mL two-neck flask under moderate stirring. As the solution started to boil slightly, 1 mL of HAuCl_4_ solution was injected. The light-yellow solution turned into a light-purple color after a few minutes and became darker during to the next 30 min. After this period, the reaction vessel was cooled down to room temperature in a water bath. The seeds started to grow after 24 h. Then, the seed solution was heated to 90 °C, under moderate stirring, and 1 mL of HAuCl_4_ solution was injected. After 30 min, 1 mL of HAuCl_4_ solution was again injected, and 30 min after the last injection, 55 mL of the solution was extracted. The remaining solution was diluted by adding 53 mL water and 2 mL of a sodium citrate solution (60 mM). The diluted solution was used as the seed solution in the next step. Each step included three times the injection of 1 mL HAuCl_4_ solution after every 30 min of reaction time followed by the extraction and dilution. For the whole syntheses, 10 steps were carried out. After the fifth step, the synthesis was stopped and cooled down to room temperature. The synthesis was continued the next day. The solution turned from dark-purple to burgundy after four steps and became cloudy. After the sixth step, a slight brown/yellowish change of color was recognized.

The extracted volumes and the final nanoparticles were stored in glass vessels in the fridge until further use. The nanoparticles of each step were investigated by UV–vis and dynamic light scattering (DLS).

UV–vis measurements of the AuNPs dispersions were carried out using the Cary 100 Bio UV–visible Spectrophotometer from Agilent Technologies (Santa Clara, California, U.S.A.). The spectral range from 300 to 700 nm was investigated and measured in one nanometer steps. As a reference for the measurements, water was used. The as-prepared dispersions of gold nanoparticles were measured right after the synthesis and one month after preparation. For the measurements, 0.5 mL of AuNP dispersion was diluted in 1.5 mL water (1:3). The samples were in the form of dispersed particles in liquid solution. Dynamic light scattering (DLS) NanoBrook Omni Zeta Potential Analyzer (from Brookhaven Instruments (Holtsville, New York, USA)) was employed for particle size measurements of the synthesized gold nanoparticles. The as-prepared dispersions were measured right after the synthesis. For the measurements 0.5 mL AuNP dispersion were diluted in 1.5 mL water (1:3 v/v). The samples were in the form of dispersed particles in liquid solution.

### 2.3 Plasma Treatment and Immobilization of AuNPs in PP Surface with the Raman Reporter (PP@AuNPs/4-MB)

PP-LD@AuNPs and PP-MD@AuNPs samples were obtained by covalent immobilization of the AuNPs with ethylenediamine (ED), following the method reported by Quidant and co-workers.^29^ However, for the polymer yarns’ activation with plasma, we followed our own optimized conditions reported elsewhere.^30^ In the first step, the meshes (PP-LD or PP-MD) were first activated with oxygen plasma (plasma power 250 W, purging pressure of 0.07 mbar, gas flow fixed for 180 seconds and 20 sccm). Plasma treatment was realized with a low-pressure radio frequency (RF) plasma (80 MHz), by using a LFG generator 1000 W (Diener Electronic GmbH Co., Germany) and a chamber of 2.5 dm^3^. Activation of PP-LD or PP-MD by plasma gives rise to O-free radical groups able to react with ED and to generate the necessary anchor bonds for the addition of AuNPs. Then, solutions of 4-mercaptobenzonitrile (4-MB) with a molar concentration of 10^–5^ M were prepared in water. Proper volumes of gold (2 mL) and 4-MB (1 mL) solutions were mixed and incubated during 4 h with the polypropylene meshes to generate PP-LD@AuNPs/4-MB and PP-MD@AuNPs/4-MB samples.

### 2.4 Hydrogel Grafting (PP-*g*-PNIPAAm@AuNPs/4-MB) and Evaluation of the Grafting Yield (GY)

The graft polymerization of NIPAAm on the PP mesh was carried out taking the advantage of the created active (−COOH, −COO^–^ and radical species) sites on top of the fibers after the plasma process^30^ and subsequently after the AuNPs covalent coupling. Then, samples were preweighed (surface area of 4 cm^2^), and were immersed in the solution containing the acrylamide monomers, the initiator and the catalyst. For experimental details, the reader can also refer to our previous references.^31,32^ After 1 h of reaction at 30 °C, the resulting samples (PP-LD-*g*-PNIPAAm@AuNPs/4-MB and PP-MD-*g*-PNIPAAm@AuNPs/4-MB) were purified onto 400 mL of Milli-Q water under stirring during 4 h, by continuous replacement of Milli-Q water and then dried at 30 °C overnight under vacuum.

The grafting yield was calculated from the difference of mesh weight and the complete device per unit area, obtaining the amount of hydrogel grafted per unit area (eq 1):
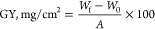
1where *W*_f_ and *W*_0_ are the final and initial weights in mg after and before grafting, respectively, and *A* is the area of the mesh in cm^2^.

### 2.5 Acquisition of Raman Spectra and SERS Enhancement Factor (SERS EF) Calculations

Raman spectra were acquired using a Renishaw dispersive Raman microscope spectrometer (model InVia Qontor, GmbH, Germany) and Renishaw WiRE software. The spectrometer is equipped with a Leica DM2700 M optical microscope, a thermo-electrically cooled charge-coupled device (CCD) detector (−70 1C, 1024 × 256 pixels) and a spectrograph scattered light with 2400 lines mm^–1^ or 1200 lines mm^–1^ of grating. The experiments were performed with two different sources, at 532 and 785 nm excitation wavelengths and with a nominal laser power between 1 and 100 mW output power. The exposure time was 10 s, the laser power was adjusted to 1% of its nominal output power, and each spectrum was collected with three accumulations. All Raman spectra were collected in a spectral range from 600 to 4000 cm^–1^ with the same measurement parameters.

The SERS EF was then determined as shown below, following the literature procedure:^33−35^
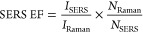
2where *I*_SERS_ and *I*_Raman_ are Raman signal intensities of 4-MB at 2230 cm^–1^ with and without SERS from AuNPs; and *N*_Raman_ and *N*_SERS_ are the number of 4-MB molecules in bulk solution being deposited on meshes without and with AuNPs within laser spot, respectively. Assuming uniform adsorption of 4-MB molecules on AuNPs and of gold nanoparticles on meshes surface *N*_*Raman*_ = *N*_*SERS*_. More in detail, *N*_*Raman*_ ≈ 0.42 × 10^12^ and 0.84 × 10^12^ 4-MB molecules were irradiated by the laser during Raman acquisition using the 532 and 785 nm lasers, respectively (see ESI for details on the derivation of those values). Taking into account that *N*_*Raman*_ = *N*_*SERS*__,_eq 2 can be simplified as follows:
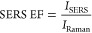
3

### 2.6 Characterization Techniques

Fourier transform infrared spectroscopy (FTIR) was carried out with a Jasco 4100 spectrophotometer to observe the main absorption bands of the hydrogel. An attenuated total reflection accessory with a diamond crystal (Specac model MKII Golden Gate Heated Single Reflection Diamond ATR) was used to place samples. A total of 64 scans were performed between 4000 and 600 cm^–1^ for each sample with a resolution of 4 cm^–1^. Scanning electron microscopy (SEM) was carried out using a Focused Ion Beam Zeiss Neon40 scanning electron microscope equipped with an energy dispersive X-ray analysis (EDX) spectroscopy system and operating at 5 kV. SEM was used to examine the internal morphology through cross-section study using cryofracture on freezed-dried samples. The meshes (surface area of 1 × 1 cm^2^) were mounted on a double-side adhesive carbon disc and sputter-coated with a thin layer of carbon to prevent sample charging problems.

X-ray photoelectron spectroscopy (XPS) analyses were used for the detection of gold nanoparticles and Raman reporter molecules. It was carried out on samples modified with different metal particles size as well as in the presence and in the absence of the TSH. The assays were performed on a SPECS system equipped with an Al anode XR50 source operating at 150 mW and a Phoibos MCD-9 detector. The pressure in the analysis chamber was always below 10^–7^ Pa. The pass energy of the hemispherical analyzer was set at 25 eV and the energy step was set at 0.1 eV. Data processing was performed with the Casa XPS program (Casa Software Ltd., U.K.).

Contact-angle measurements were carried out using the water sessile drop method. Images of 0.5 μL distillated water drops were recorded after stabilization with the equipment OCA 15EC (Data-Physics Instruments GmbH, Filderstadt). SCA20 software was used to analyze the images and determine the contact angle value, which was obtained as the average of at least 10 independent measures for each sample. Before the analysis, the samples were fixed in a metallic support, and for temperature evaluation, they were heated on a hot plate and moved to the equipment for measurements.

### 2.7 Mesh Unfolding Measurements under Controlled Temperature by Using an Infrared Camera

Two tests were conducted to monitor motion of PP-LD @AuNPs/4-MB and PP-MD-*g*-PNIPAAm@AuNPs/4-MB samples in air under controlled humidity conditions. The samples were previously swollen in water and dried overnight in the folded conformation, in a temperature higher than the LCST of the hydrogel (32–33 °C) to ensure the contraction of the PNIPAAm chains (40 °C in an oven).

Samples were placed in a controlled humidity chamber (relative humidity of 100%), and an infrared (IR) imaging camera (Optris PI connect 640) was installed at 23 cm distance from the chamber to record sequential images of the scene. The spectral range of the IR camera used is from 7.5 to 13 μm, and the frame rate used in this work was of 1 Hz. The Optris PIX Connect software was used to set display and recording options, and a Python script was implemented to process afterward data records. This script was programmed to obtain median temperature values in a small area containing the biomedical prosthesis of interest and to calculate the unfolding angle (θ). This angle was obtained using a mouse call-back function implemented in Python to manually select the tip of the sample on each frame. Trigonometric ratios were used then to calculate the angle according to the position.

Since temperatures reported in this work were measured using a noncontact method, i.e. a thermal IR camera, it is important to take into account that the electric signal of a detector (*U*) in this kind of equipment is related to the temperature of the body (*T*_object_) according to the Stefan–Boltzmann law (eq 4):
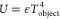
4where ε refers to the emissivity of the body.

Therefore, to precisely determine the temperature of a body by using a thermal IR camera, the emissivity of the body under study is essential. In the present work, an emissivity value equal to 1 was used as an approximation because the materials present in the mesh showed very different emissivities. Metal particles (gold) have a very low emissivity (0.018–0.030), whereas plastics present an emissivity value almost equal to 1 (0.91–0.93). This implies that temperatures reported in this work refer to brightness temperatures, that is, to the temperature of a blackbody (ε = 1) that would emit the same amount of radiation as the mesh under study.

## 3 Results and Discussion

### 3.1 Functionalization of PP Meshes with AuNPs and Raman Reporter: Influence of AuNPs Size, Raman Source, and Mesh Architecture in the SERS Sensor Tag

**Figure 1:**
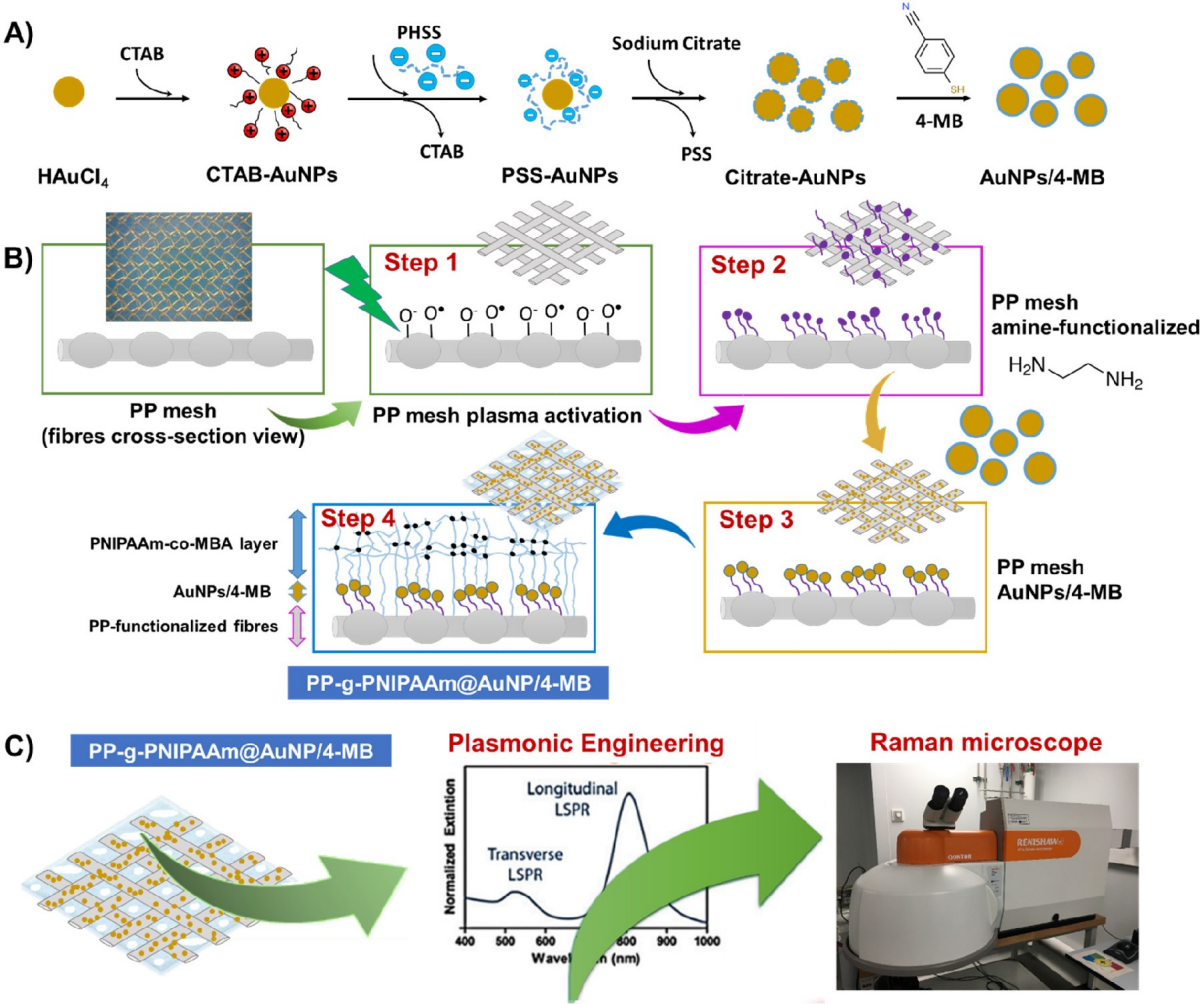
Schematic view for the preparation of a plasmon-enabled thermosensitive PP mesh material: (A) AuNPs/4-MB synthesis; (B) PP surface functionalization by plasma activation and subsequent anchoring of AuNPs/4-MB and hydrogel deposition; and (C) translation of functionalized mesh material to Raman microscope, used to evaluate the SERS effect.

**Figure 2:**
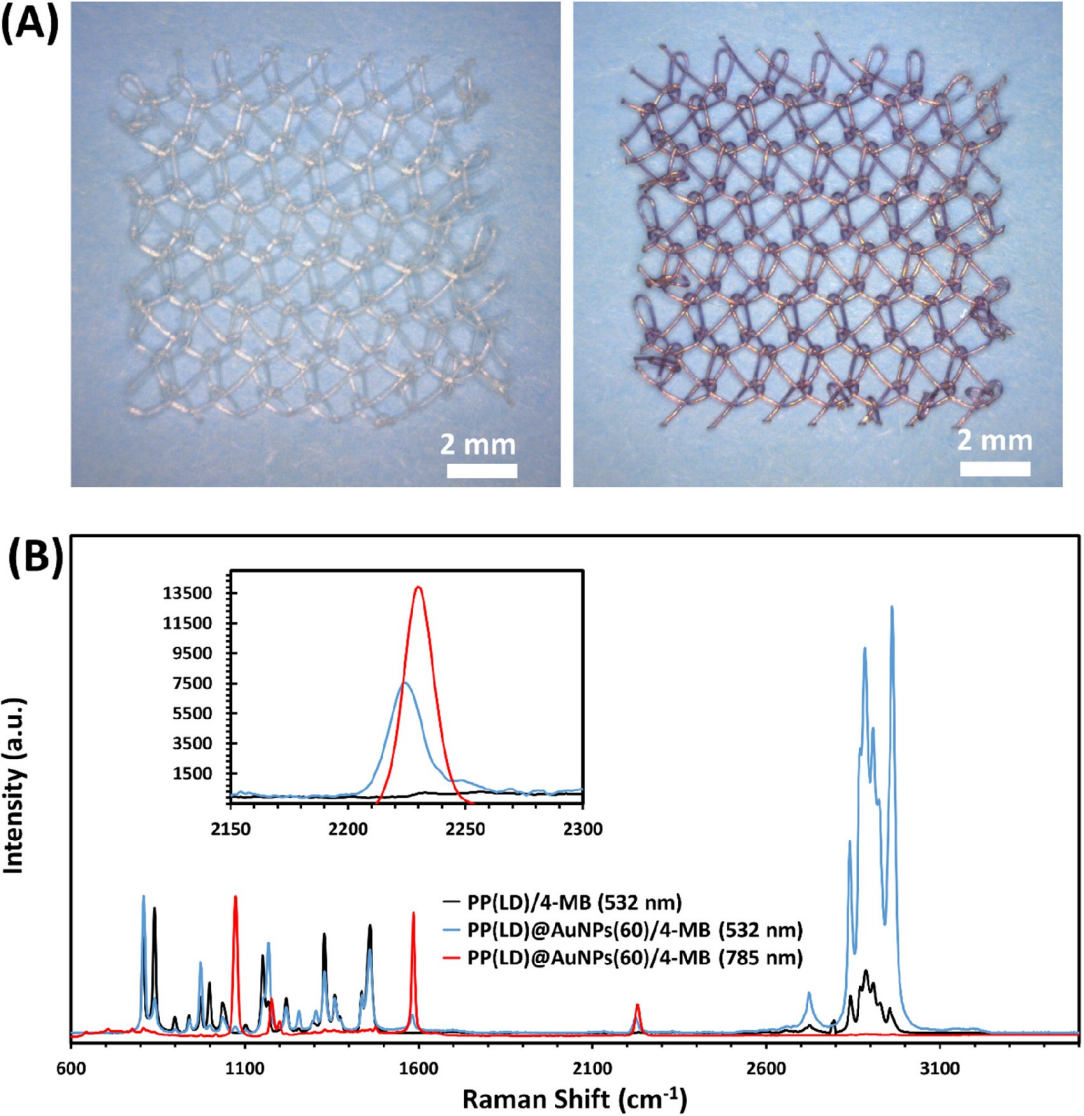
(A) Digital photographs of PP-LD mesh (low density), before (left) and after (right) covalent bonding of AuNPs/4-MB; and (B) Raman spectra of PP-LD and PP-LD@AuNPs/4-MB meshes, the particle size average of AuNPs determined by DLS being 59.0 ± 0.1 nm. The Raman spectra were recorded with two different laser sources. The inset image reflects the intensity of the C≡N absorption band of 4-MB at ∼2230 cm^–1^.

The main steps of AuNPs and 4-MB synthesis, mesh conjugation, and hydrogel deposition, as well as the schematic representation of SERS analyses, are shown in Figure 1. As previously defined, the main objective of the study is the design of a new polypropylene mesh SERS and thermally sensitive by surface bonding with AuNPs/4-MB (Figure 1A), and the application of a thermosensitive hydrogel (PNIPAAm-*co*-MBA) coating. The effect on the grafting reaction of the PNIPAAm-*co*-MBA and the presence of the AuNPs/4-MB in the SERS responsive properties were studied using two different mesh architectures and pore sizes (PP-LD or PP-MD, Figures 2A and S1, respectively), as well as two different AuNP sizes (49.9 ± 0.9 nm and 59.0 ± 0.1 nm). Furthermore, the SERS activity was investigated by means of laser sources with different excitation wavelengths (532 and 785 nm).

The successful results of such first step (Figure 1B), that is, the surface functionalization, is detected by the color change of the mesh fibers (from white to purple, Figure 2A), as well as by the uniform distribution of the AuNPs on the PP-LD surface (further proved by SEM and XPS analyses, as discussed below). The AuNPs anchoring stability and its covalent immobilization on the mesh surface were confirmed by 10 consecutive washings of the PP-LD@AuNPs/4-MB samples without detection of AuNPs in the supernatant solution (UV spectroscopy). Similar results were obtained for PP-MD@AuNPs/4-MB (Figure S1A).

After the deposition of the reporter molecules (4-MB), Raman measurements were carried out on both PP-LD/4-MB and PP-LD@AuNPs/4-MB samples. The last was modified with AuNPs with a diameter of 59.0 ± 0.1 nm and a surface plasmon resonance (SPR) of ∼530 nm. In Figure 2B, the characteristic peaks of PP-LD and 4-MB were observed. Stretching vibrations of the C–C, −CH_2_, and −CH_3_ main chain bonds of isotactic polypropylene (between 800 and 1500 cm^–1^) and the peaks due to stretching vibrations of the – CH_2_– and – CH_3_ linkages (2600 to 3000 cm^–1^) are shown.^36,37^ The Raman reporter was selected considering its strong nitrile group (C≡N) located at 2230 cm^–1^, easy to be detected and distinguished from peaks assigned to PP-LD.^38^ Taking into account the values of the SPR of the gold nanoparticles solutions, two sources for Raman excitation wavelengths of 532 and 785 nm were selected. The inset of Figure 2B clearly shows that the presence of the AuNPs is essential for the detection of the Raman reporter molecules, with 22 being the SERS EF which is calculated by comparing the signal at ∼2230 cm^–1^ in the absence and in the presence of AuNPs, with the green source (532 nm). The utilization of the NIR laser (785 nm) led to further improvement of the SERS EF in the PP-LD@AuNPs/4-MB SERS tags, that is, doubling the intensity related to the peak of the reporter molecule and increasing the SERS EF from 22 to 36.

In order to analyze the influence of mesh architecture on the SERS properties, Raman measurements were carried out also on midweight polypropylene mesh modified with AuNPs/4-MB (PP-MD@AuNPs/4-MB). Both meshes, light-and midweight, were covered by AuNPs with a diameter of 59.0 ± 0.1 nm. PP-MD@AuNPs/4-MB present similar results to PP-LD@AuNPs/4-MB in terms of both good distribution of the gold nanoparticles on the PP fibers surface (Figure S1A) and Raman intensity of the 4-MB peak at 2230 cm^–1^ (Figure S1B) with a SERS EF of 21, similar to the lightweight mesh (SERS EF of 22).

As largely reported,^39−41^ the size of the nanoparticles is a crucial parameter that could affect the surface plasmon resonance. Some authors reported that using the largest possible nanoparticle size will produce the highest SERS signal, but only changing the nanoparticle size is not an efficient way to boost the SERS signal.^42^ Not only the dimensions of the nanoparticles but also the accumulation on the substrate and the aggregation seems to affect the performance. By tuning the plasmonic coupling and the nanoparticle surface coverage, the SERS performance for the ultrasensitive detection of molecules can be optimized.^43^

**Figure 3:**
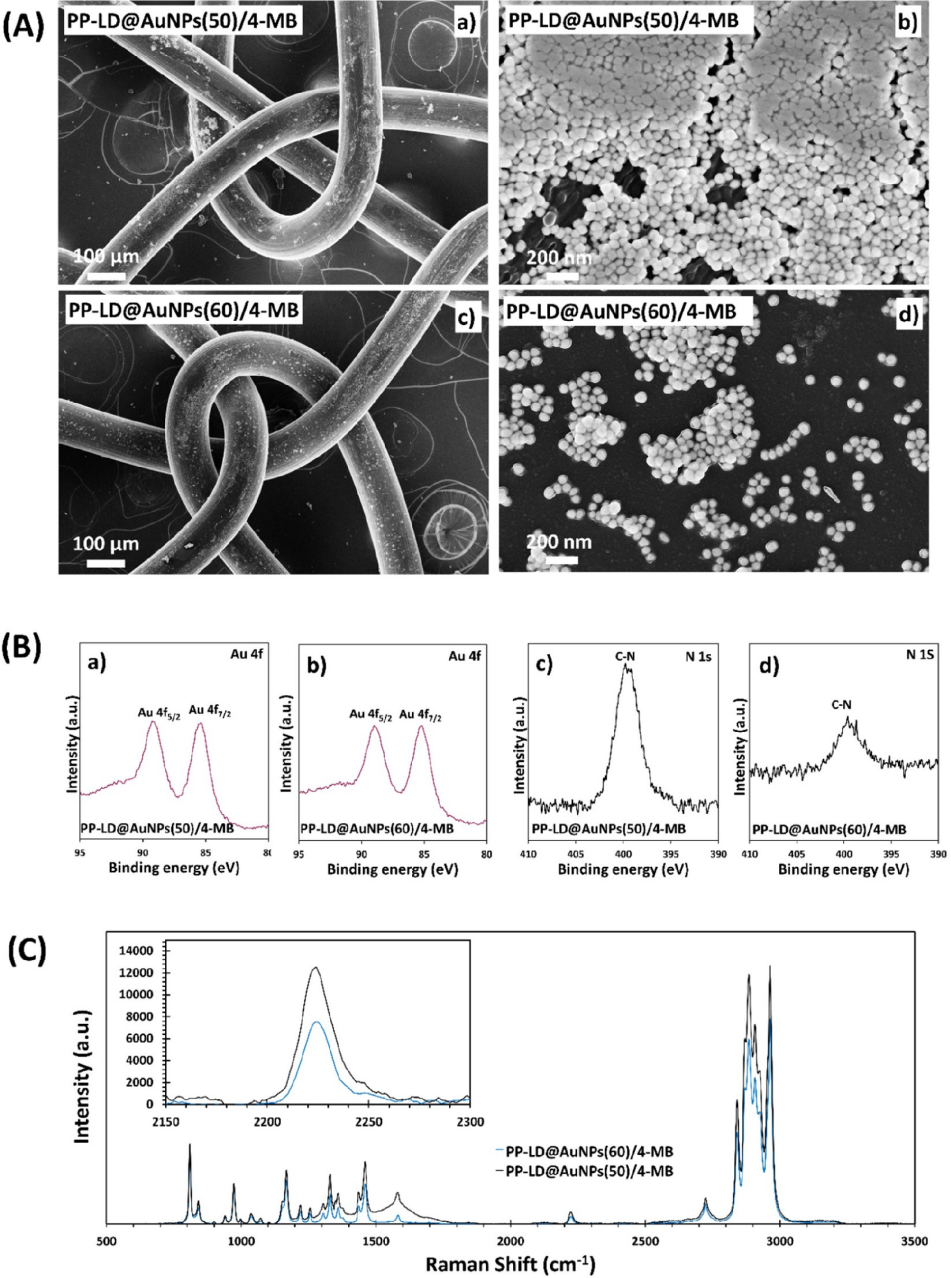
(A) SEM micrographs showing the distribution of AuNPs/4-MB in PP-LD fibers: (a,b) low- and high-magnification images with AuNPs of 50 nm and (c,d) low- and high-magnification images with AuNPs of 60 nm. (B) XPS high-resolution spectra of PP-LD fibers: (a,b) Au 4f 5/2 and 7/2 binding energies; and (c,d) N 1s binding energy, for AuNPs of 50 and 60 nm, respectively. (C) Raman spectra of PP-LD@AuNPs/4-MB with variable Au particle sizes. The inset image reflects the intensity of C≡N absorption band of 4-MB at 2230 cm^–1^. The laser source used was 532 nm.

In this work, SEM micrographs carried out on PP-LD@AuNPs/4-MB covered by AuNPs with a diameter of 49.9 ± 0.9 nm and 59.0 ± 0.1 nm (herein those NPs sizes will be reported as 50 and 60 nm for simplification) are reported in Figure 3A. A different distribution of the NPs above the PP fibers was found, being more uniform when the smallest AuNPs were used, whereas the presence of agglomerates were found for the biggest AuNPs. The different surface coverage could be attributed to the interaction of the different sized NPs with the active sites created by plasma treatment and the subsequent functionalization with ethylenediamine. In order to better understand if the amount of Au and 4-MB molecules, covering the mesh surface, is affected by the dimension of the metal particles, XPS measurements were carried out. Figure 3B displays the high-resolution XPS spectra of Au 4f_7/2_ and Au 4f_5/2_ (85 and 88 eV BE, respectively) and N 1s elements collected for PP-LD@AuNPs/4-MB covered by AuNPs of diameter 50 and 60 nm. The Au 4f_7/2_ BE is located at 85 eV,^44^ which is higher than for bulk Au(0) (83.8 eV).^45^ This could be attributed to partially charged Au^δ+^ species in the citrate stabilized sample.^46^ Binding energies typical of pairs related to the only two stable gold oxidation states Au^1+^ are slightly higher than the ones observed (BEs of 85.6 and 89.1 eV), confirming that the species detected are complexes of gold and citrate.^47^ XPS was also employed to investigate the N 1s core-levels of the 4-MB molecules employed as Raman reporter (RaR) substance. The high-resolution N 1s peak at 398.2 eV is attributed to chemisorbed benzonitrile, whose intensity increased in PP-LD@AuNPs/4-MB samples covered by AuNPs of diameter 50 nm (Figure 3B).^48^ This confirms that higher amount of 4-MB was immobilized in the sample with the smallest AuNPs size, corroborating SEM observations (Figure 3A (a,b)), in which a greater quantity and distribution of nanoparticles is observed through the PP fibers of the PP-LD@AuNPs/4-MB sample.

SERS spectra reported by Figure 3C are in good agreement with the results observed in Figure 3A,B. The intensity of the Raman signal increases when PP-LD@AuNPs/4-MB samples are covered by the smallest AuNPs, confirming that the accumulation on the substrate is crucial to cause the plasmonic coupling and improve the SERS detection.

### 3.2 Effect of the Presence of PNIPAAm-*co*-MBA in the SERS Sensor Tag Detection

**Figure 4:**
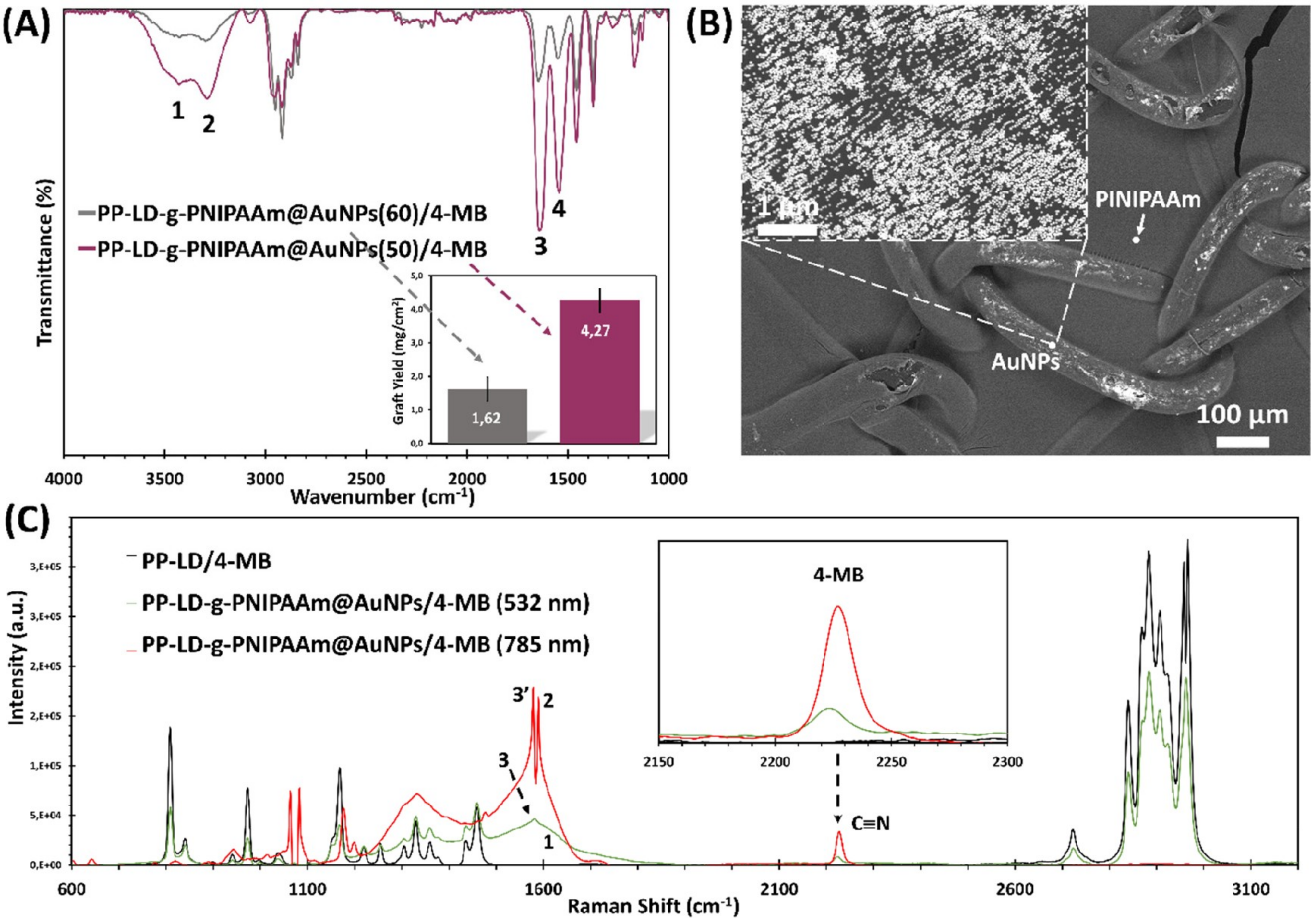
(A) FTIR spectra of PP-LD-*g*-PNIPAAm@AuNPs/4-MB with AuNPs average diameters of 50 and 60 nm. The inset represents the GY values of both samples. (B) SEM micrograph of PP-LD fibers functionalized with AuNPs/4-MB particles and a coat of PNIPAAm-*co*-MBA hydrogel. A backscattering detector was used to be able to differentiate the metallic particles and the polymer coating. (C) Raman spectra of PP-LD-*g*-PNIPAAm@AuNPs/4-MB showing the increasing of the C≡N absorption band (inset image) at ∼2230 cm^–1^ with lasers of 532 and 785 nm.

The effect of the nanoparticles size on the grafting of the thermosensitive hydrogel was first studied in this work by means of FTIR and Raman measurements. In the first case, the resulting FTIR spectrum (Figure 4A) shows the characteristic peaks of PNIPAAm in PP-LD-*g*-PNIPAAm@AuNPs/4-MB sample covered by both 50 and 60 nm sized AuNPs. The functional groups assigned to the thermosensitive hydrogel correspond to the bands located in the range of 3200 to 3600 cm^–1^ related to −OH groups (**1**) and −NH_2_ vibrations (**2**), respectively, and the peaks of the C=O stretching of amide I (1640 cm^–1^, **3**) and of the NH bending of amide II (1540 cm^–1^, **4**).^49^ The inset of Figure 4A shows the GY obtained, which increases from 1.62 ± 0.4 to 4.27 ± 0.4 mg/cm^2^ by varying the AuNPs size (from 60 to 50 nm).

Surprisingly, an effect of AuNPs size and distribution above the PP yarns that affects the PNIPAAm network was observed. Close inspection of SEM images (Figure 4B) shows the homogeneous distribution of the PNIPAAm coating as well as of AuNPs on the surface of the mesh fibers. From these results, it is possible to conclude that the PNIPAAm formation is facilitated in the presence of the smallest (50 nm of diameter) and better distributed AuNPs, which explains the fact the GY is more than twice in this case.

In order to ensure the AuNPs anchoring stability after the hydrogel grafting, release studies by UV–vis light of AuNPs, in phosphate buffer solution (PBS) at 37 °C, were carried out in the presence of PP-MD@AuNPs/4-MB (i.e., without hydrogel) and PP-MD-*g*-PNIPAAm@AuNPs/4-MB meshes (i.e., with the hydrogel protective layer). The typical absorbance band of the AuNPs centered at ∼530 nm (Figure S2A) were not detected, either in in the absence (Figure S2B) or in the presence (Figure S2C) of a hydrogel layer. The leaching study was performed during samples’ immersion in 1, 2, 4, 8, 24 and 48 h, confirming the strong covalent bonds of the AuNPs with the PP matrix.

Figure 4C reports SERS spectra recorded in the presence of PNIPAAm hydrogel grafted on a mesh surface that was previously functionalized with 50 nm sized AuNPs and 4-MB as RaR molecules. The strategy developed in the present work to create the PP-LD-*g*-PNIPAAm@AuNPs/4-MB platform for Raman detection has proved to be successful even if a thick layer of hydrogel is covering the PP gold-functionalized substrate. The Raman spectrum of the sample without AuNPs only shows the absorption bands of the plastic, being impossible to find any 4-MB peak, as expected. After covalent immobilization of AuNPs/4-MB with ethylenediamine, the presence of such a small molecule is detected as a broad band (**1**) at around 1600 cm^–1^, which appears overlapped with the amide I peak (**2**) of PNIPAAm.^50^ However, the presence of such a band (**1**) does not difficult the localization of the absorption bands from benzene rings of 4-MB molecules. The C=C–Ar ring vibrations appear as a small peak (**3**), when using a short-wavelength laser (532 nm), and as highly intense and strong peak at 1587 cm^–1^ (**3′**), besides the Amide I of PNIPAAm-*co*-MBA hydrogel. This strong peak is in accordance to previous studies,^51^ corroborating that 4-MB is an useful RaR for our biomedical platform. Moreover, the presence of such sensor molecules is further confirmed by the peaks localized at 2230 cm^–1^, corresponding to the C≡N groups. Finally, inspection of the SERS intensity (eq 3) only reveals a slight reduction of the SERS EF from 36 to 24 (Table 1) when samples without (PP-LD@AuNPs(50)/4-MB) and with the TSH coating (PP-LD-*g*-PNIPAAm@AuNPs(50)/4-MB) are compared (i.e., the same particle size and laser excitation was used for both cases).

**Table 1:** SERS Enhancement Factor (EF), Calculated from Equation 3, at Different Conditions

sample	AuNPs diameter (nm ± SD)	laser wavenumber (nm)	SERS EF
PP-LD/4-MB	-	532	**1**
PP-LD@AuNPs(60)/4-MB	58.98 ± 0.05	532	**22**
PP-LD@AuNPs(50)/4-MB	49.86 ± 0.87	532	**36**
PP-LD-g-PNIPAAm@AuNPs(50)/4-MB	49.86 ± 0.87	532	**24**
PP-LD-g-PNIPAAm@AuNPs(50)/4-MB	49.86 ± 0.87	785	**96**

Looking into the search for the best laser source to detect 4-MB RaRs in the SERS mesh modified surfaces, we compared the SERS response of the PP-LD-*g*-PNIPAAm@AuNPs(50)/4-MB sample with two lasers of 532 and 785 nm. A strong increase of the intensity of the RaR molecules was achieved (inset of Figure 4C), with the longest wavelength employed (785 nm), resulting in a SERS EF of 96. The latter could be ascribed to the higher penetration power of the IR laser that allow reaching more molecules of 4-MB under the PNIPAAm layer, without damage the gel layer. Table 1 reports the values of SERS EF obtained at different operating conditions and using different AuNPs diameter and lasers.

The results shown in Table 1 prove that SERS response can be easily scalable to other AuNPs sizes. Lower NPs diameters can be translated in a higher Raman intensity, even with a layer of PNIPAAm-co-MBA hydrogel covering such SERS particles.

Regarding the prosthesis architecture, the grafting yield (GY) of the TSH varied when the distribution of PP fibers changes from low-density (LD) to medium-density (MD) (Figures 2A and S1A). As was proved by FTIR spectroscopy (Figure S3A), the low density (PP-LD) and the medium density (PP-MD) polypropylene meshes were both well covered with the TSH. The GY increased from 1.624 ± 0.35 to 3.561 ± 0.32 mg/cm^2^ (Figure S3B) when the density of the PP mesh increased. This effect cannot be attributed to the particles size itself, but rather to the knitted configuration of the PP-MD mesh, which has larger pores that favors gel aggregation among PP yarns. On the contrary, the distribution of the AuNPs was deficient (not uniform) in PP-MD substrates, as observed by SEM (Figure S3C) and even macroscopic photographs (Figure S1B). As reported in the literature,^52^ increasing the particle size leads to decrease particle coverage, affecting the localized surface plasmon resonance, that is, making worse the SERS activity of individual AuNPs. Furthermore, SERS EF monotonically enhances when the separation distance between particles in a dimer diminishes.^52^ On that basis, the uniform distribution of the gold nanoparticles achieved in this work with the PP-LD@AuNPs(50)/4-MB platform, ensures not only the minimal distance between the particles but also the conservation of particle size, both properties being beneficial for the SERS activity. For this reason, the PP-LD-g-PNIPAAm@AuNPs/4-MB was selected as the optimized platform for the final biomedical sensor buildout.

### 3.3 How the Presence of AuNPs/4-MB and Mesh Knitted Configuration Can Affect the Stimulus Responsive Behavior of the PNIPAAm-co-MBA Thermosensitive Hydrogel Adhered to the Mesh Fibers?

After proving that nonabsorbable biomedical meshes functionalized with AuNPs and 4-MB fingerprint molecules can be detected by SERS, we finally followed-up the effect of such particles (50 nm of particle size only) on PP-g-PNIPAAm thermosensitive response. It was performed by monitoring: (i) the hydrophilicity/hydrophobicity behavior of the PNIPAAm grafted to PP meshes with water contact angles (θ) measurements (Figure S4); and (ii) the macroscopic folding-unfolding movement of two biomedical prosthesis with thermal IR imaging under high humidity conditions.

The wettability of PP-LD-g-PNIPAAm@AuNPs/4-MB was investigated. More in detail, contact angles were collected at 25 °C in dry and wet conditions and at 38 °C in wet conditions (Figure S4). The contact angle decreased from the dry to the wet mesh (from θ = 50.5 ± 1.0° to θ = 14.6 ± 0.8°) and increased significantly when the temperature is higher than the copolymer’s LCST (from θ = 14.6 ± 0.84° to θ = 30.4 ± 1.1°). In both cases, the increase in hydrophobicity of the biomedical device after LCST is ascribable to the pore closure of the PNIPAAm hydrogel, which expels the water when the hydrogel chains contract.

Successive heating and cooling cycles were performed in a humidity chamber to monitor the behavior of the samples (4D response)^32^ in response to the thermal stimulus. IR temperatures (*T*_IR_) ranged from 25.0 to 45.0 °C.

**Figure 5:**
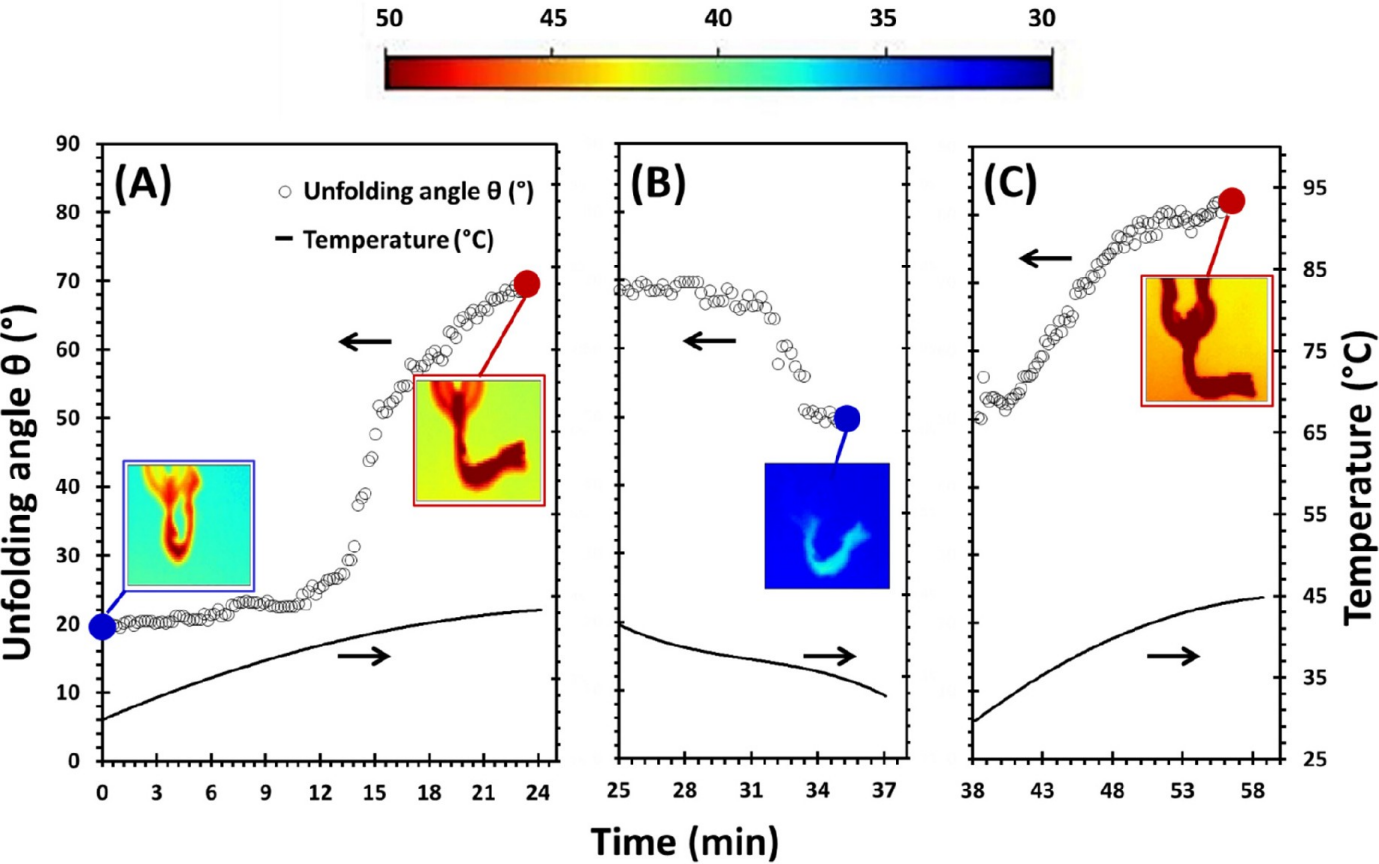
Variation of unfolding angles and temperature versus time for PP-LD-g-PNIPAAm@AuNPs/4-MB under cooling and heating cyclic experiments: (A) first heating, (B) first cooling, and (C) second heating. The insets are infrared (IR) images of the modified mesh at the corresponding values of temperature and unfolding angles represented by blue and red dots. The colorful bar shows the temperature distribution. The color bar on top provides a key for the meaning of colors representing temperatures in IR images.

Figure 5 illustrates the progressive thermo-induced opening/folding of the bilayer PP-LD-g-PNIPAAm@AuNPs/4-MB mesh and the corresponding values of unfolding angle (*y*-axis on the left side) under temperature (*y*-axis on the right side) changes. When the sample is placed in the chamber with controlled humidity and increasing temperatures, starting by its initial dried state and with an initial opening fold of 20° (at 19.0 °C), the mesh begins to unfold at *T*_IR_ = 37.0 °C. An unfolding angle of 50° is achieved after 12 min during the first heating step at *T*_IR_ = 45.0 °C (Figure 5A). Consecutive steps of cooling and heating were applied, showing a quasi-reversible motion of PP-LD-g-PNIPAAm@AuNPs/4-MB with a folding (closure of the mesh) of θ = 20° during the cooling step (Figure 5B) and a consecutive unfolding of θ = 35° during the second heating step (Figure 5C). Taking into account that the LCST of the PP-LD-g-PNIPAAm is about 33.5 °C, as reported in our previous study,^32^ it was expected to observe this motion in the range of 32–35 °C. The higher temperature observed (*T*_IR_ = 37 °C) is ascribable to the heat generation by plasmon-resonant gold NPs present in the PP yarns. As stated previously, infrared temperatures reported here (*T*_IR_) refer to a set emissivity equal to 1 for the emitting object (i.e., the mesh). Therefore, since the emissivity of Au is very low, actual temperatures of the mesh measured with the IR camera provide a reliable representation of the whole system.

For the sake of comparison, a PP-LD mesh functionalized with AuNPs/4-MB without TSH was also investigated. Figure S5 shows that the unfolding movement is not thermally controlled, as occurs with the PP-LD-g-PNINPAAm@AuNPs/4-MB mesh (Figure 5). Actually, the unfolding angle observed during the first heating step (θ = 50° at 22 min, Figure S5A) might be attributed only to the increased weight of the mesh when aqueous vapor water molecules deposit on its surface. There is no reversible folding-unfolding angle with temperature variations during the successive cooling and heating cycles, that is, the unfolding angle was maintained constant for 33 min of assay (Figures S5B,C). Altogether, this confirms that PP-LD functionalized with AuNPs is not thermoresponsive and the effect of unfolding is due to the mesh movement under gravity.

In summary, the presence of AuNPs impacts on the thermal responsiveness of the whole system, affecting the LCST temperature of the hydrogel as recorded using thermal IR imaging. However, AuNPs help to enhance the thermosensitivity stability and cyclic behavior of the whole platform when low-density knitted substrates are employed. This response (temperature sensitivity about 37 °C) is ideal for the development of an implantable sensor, which enables self-folding and unfolding inside the body if local inflammatory processes appears (caused by fever or local inflammation). With localized AuNPs, SERS detection and semi-invasive techniques (e.g., by using laparoscope technique), the finding of inflammatory causes could be evidenced by the localization of the real position of the mesh once implanted.

## 4 Conclusions

A successful methodology to convert a polypropylene surgical mesh knitted structure into a thermosensitive and SERS responsive surface has been described. The incorporation of AuNPs and PNIPAAm hydrogel allowed the detection of temperature changes that are similar to the body temperatures associated to inflammatory processes in operated patients (postsurgery problems). By using Raman spectroscopy, it was possible to clearly identify the RaR molecules trapped above the AuNPs and mesh surface, even with a thick layer of PNIPAAm hydrogel. Moreover, by using an IR imaging camera, it was possible to check the temperature changes above the mesh, when experiencing folding-unfolding apertures provoked by local temperature and humidity conditions.

The smallest AuNPs and the low-density biomedical textile facilitated the covalent bonding of the PNIPAAm hydrogel because of the well-distributed AuNPs/4-MB spherical particles over the PP yarns, as proved by SEM images. Moreover, the thermal stimulus response of the coated samples was certified by IR imaging, showing also a reversible effect.

Furthermore, the adaptation of such innovative polymer surface sensors, in the future, to other inanimate biomedical plastics (e.g. surgical sutures, grapes, wounds, and others, fabricated with nonabsorbable polymers) can open an efficient prevention tool against recurrent outbreaks of clinical interventions by using semi-invasive additional tools.

## Supporting Information Available

Detailed procedure for the normalization of SERS intensity with Raman reporter molecules; representative images of meshes, before and after modification with AuNPs, and Raman spectra (Figure S1); UV–vis spectra of AuNPs solutions with different particle sizes and of PBS solutions during AuNPs release studies (Figure S2); FTIR spectra, graft yield, and SEM micrograph of PP-g-PNIPAAm@AuNPs/4-MB samples (Figure S3); contact angle measurements at different (Figure S4); variations of unfolding angle and temperature versus time with an infrared camera (Figure S5) (PDF)
