# Supplementary material for: Dual-Responsive Polypropylene Meshes Actuating as Thermal and SERS Sensors
Source: ACS Biomater Sci Eng. 2022 Jun 2;8(8):3329–40. doi: 10.1021/acsbiomaterials.2c00334 (PMC9988207; doi:10.1021/acsbiomaterials.2c00334)
Supplement: Supplementary file 1 — ab2c00334_si_001.pdf [file ab2c00334_si_001.pdf]

Dual-responsive polypropylene meshes actuating as thermal and SERS sensors

Sonia Lanzalaco,*[a,b] Pau Gil,[a] Júlia Mingot,[a,b] Alba Àgueda,[c] Carlos Alemán[a,b,d] and Elaine Armelin*[a,b]

[a] *Departament d'Enginyeria Química, IMEM-BRT, EEBE, Universitat Politècnica de Catalunya, C/ Eduard Maristany, 10-14, Ed. I, 2nd floor, 08019, Barcelona, Spain.*

[b] *Barcelona Research Center in Multiscale Science and Engineering, Universitat Politècnica de Catalunya, C/ Eduard Maristany, 10-14, basement S-1, 08019, Barcelona, Spain.*

[c] *Departament d'Enginyeria Química, CERTEC, EEBE, Universitat Politècnica de Catalunya, C/ Eduard Maristany, 10-14, Ed. I, 5th floor, 08019, Barcelona, Spain.*

[d] *Institute for Bioengineering of Catalonia (IBEC), The Barcelona Institute of Science and Technology, Baldiri Reixac 10-12, 08028, Barcelona, Spain.*

*Corresponding authors: E-mail: sonia.lanzalaco@upc.edu (S. Lanzalaco)

E-mail: elaine.armelin@upc.edu (E. Armelin)

EXPERIMENTAL PROCEDURE

Number of 4-MB molecules excited for Raman (N_{Raman}) and for SERS with AuNPs (N_{SERS})

Here, we provide an estimation of the number of 4-MB molecules excited by Raman (N_{Raman}) and the number of 4-MB molecules excited for SERS with AuNPs (N_{SERS}). After depositing 4-MB on the mesh substrate, the observed diameter of 4-MB spot is ~0.11 µm, giving a 4-MB spot size of 9.5×10^{-5} cm^2. For mesh substrate without AuNPs, the initial bulk contained 4-MB = 10^{-2} mM × 1 mL = 1×10^{-8} moles of 4-MB molecules. Assuming homogeneous a distribution of 4-MB molecules on mesh, the number of bulk 4-MB molecules within laser spot can be calculated from the area ratio of laser spot size to 4-MB spot size. The laser spot size considering the diffraction pattern resulting from the illuminated circular aperture of the laser irradiation. Mathematically, the diffraction pattern is characterized by the wavelength of light illuminating the circular aperture, and the aperture's size. The diameter of laser spot can be estimated by the following equation:

$$d = \frac{1.22\,\lambda}{NA} \qquad (1)$$

where λ is the laser wavelength and NA is the numerical aperture. In our study, λ = 532 or 785 nm, NA = 0.75, so d ~0.9 µm (for 532 nm) and d ~1.3 µm (for 785 nm), giving a laser spot size of 0.64 and 1.33 µm^2, respectively.

Taking into account these values, the area ratios of laser spot size (0.64 µm^2 for 532 nm and 1.33 µm^2 for 785 nm) to 4-MB spot size (9.5×10^{-5} cm^2) are 0.07×10^{-3} and 0.14×10^{-3}, respectively, which means 0.07×10^{-11} and 0.14×10^{-11}moles of 4-MB molecules, respectively using the laser with 532 nm and 785 nm as wavelength, respectively. These N_{Raman} ~0.42×10^{12} and 0.84×10^{12} 4-MB molecules are irradiated by the laser during Raman acquisition by means of 532 nm and 785 nm sources, respectively.

RESULTS

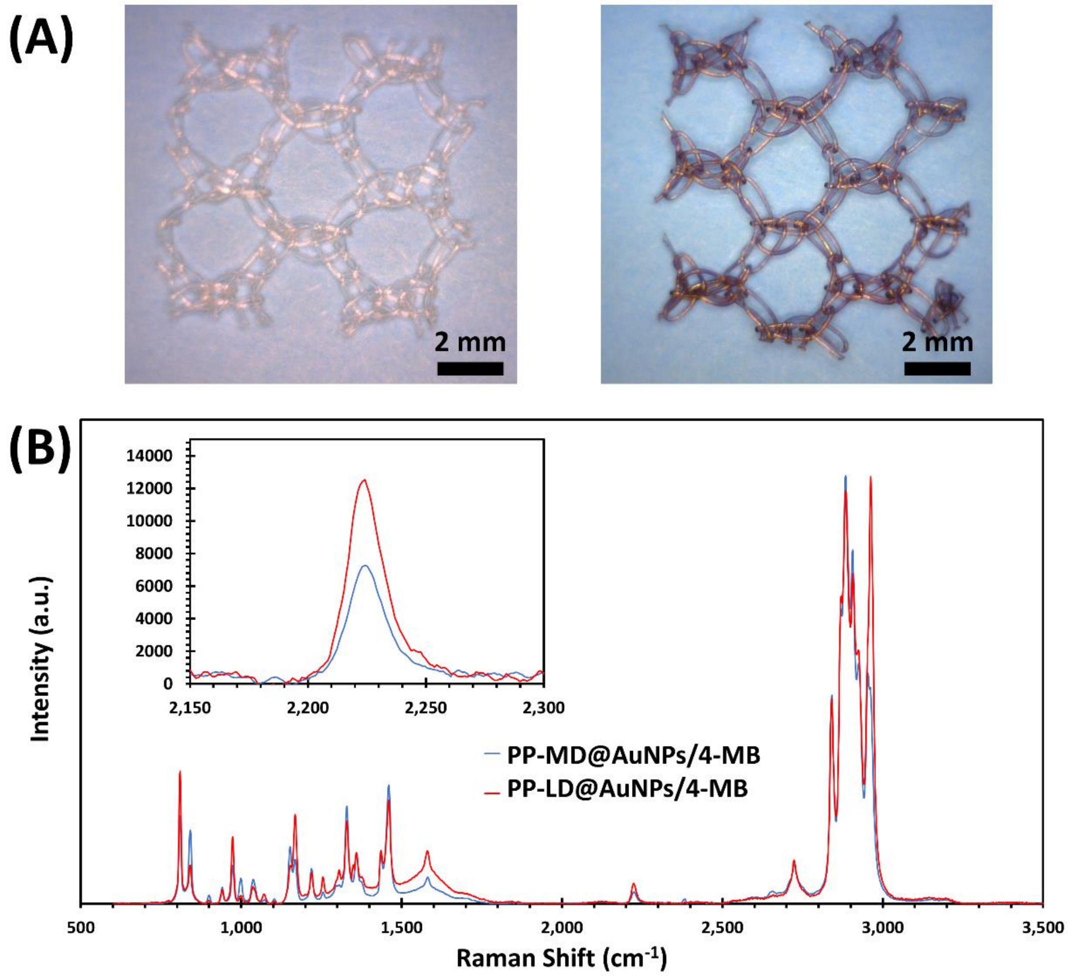

Figure S1. (A) Digital photographs of PP-MD mesh (medium density), before (left) and after (right) covalent bonding of AuNPs/4-MB; and (B) Raman spectra of PP-MD@AuNPs/4-MB and PP-LD@AuNPs/4-MB meshes, with AuNPs of 59.0 ± 0.1 nm of particle size. The Raman spectra were recorded with laser of 532 nm. Inset image reflects the intensity of C≡N absorption band of 4-MB at ~2230 cm^{-1}.

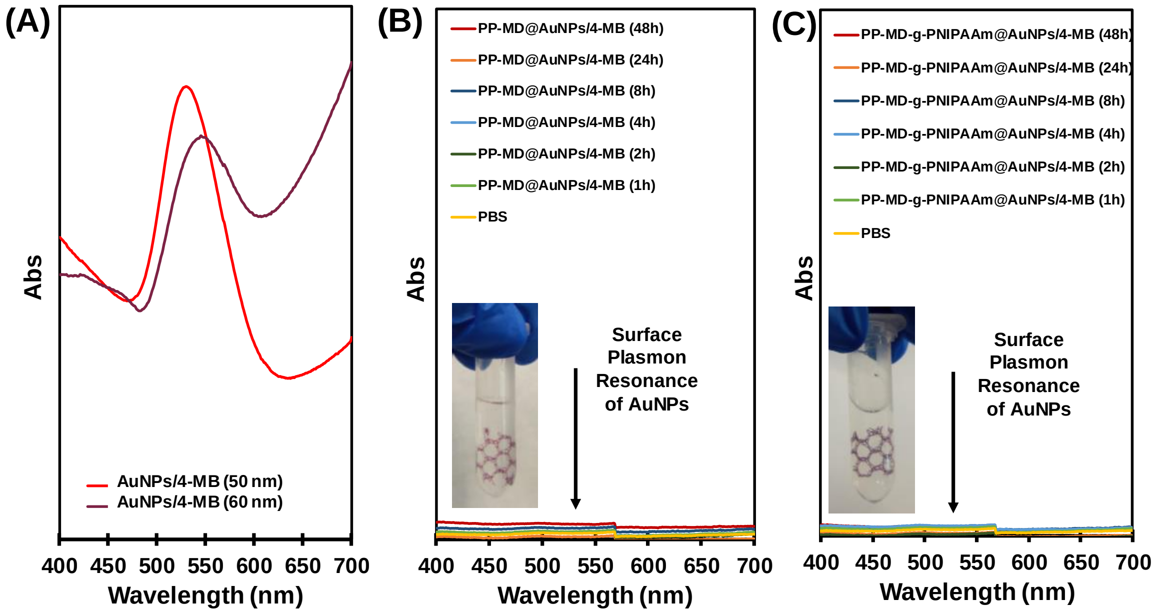

Figure S2. UV-vis spectra of: (A) AuNPs pure solutions with different particle sizes; and (B-C) PBS solutions during AuNPs release studies in the absence (B) and in the presence (C) of PNIPAAm hydrogel layer.

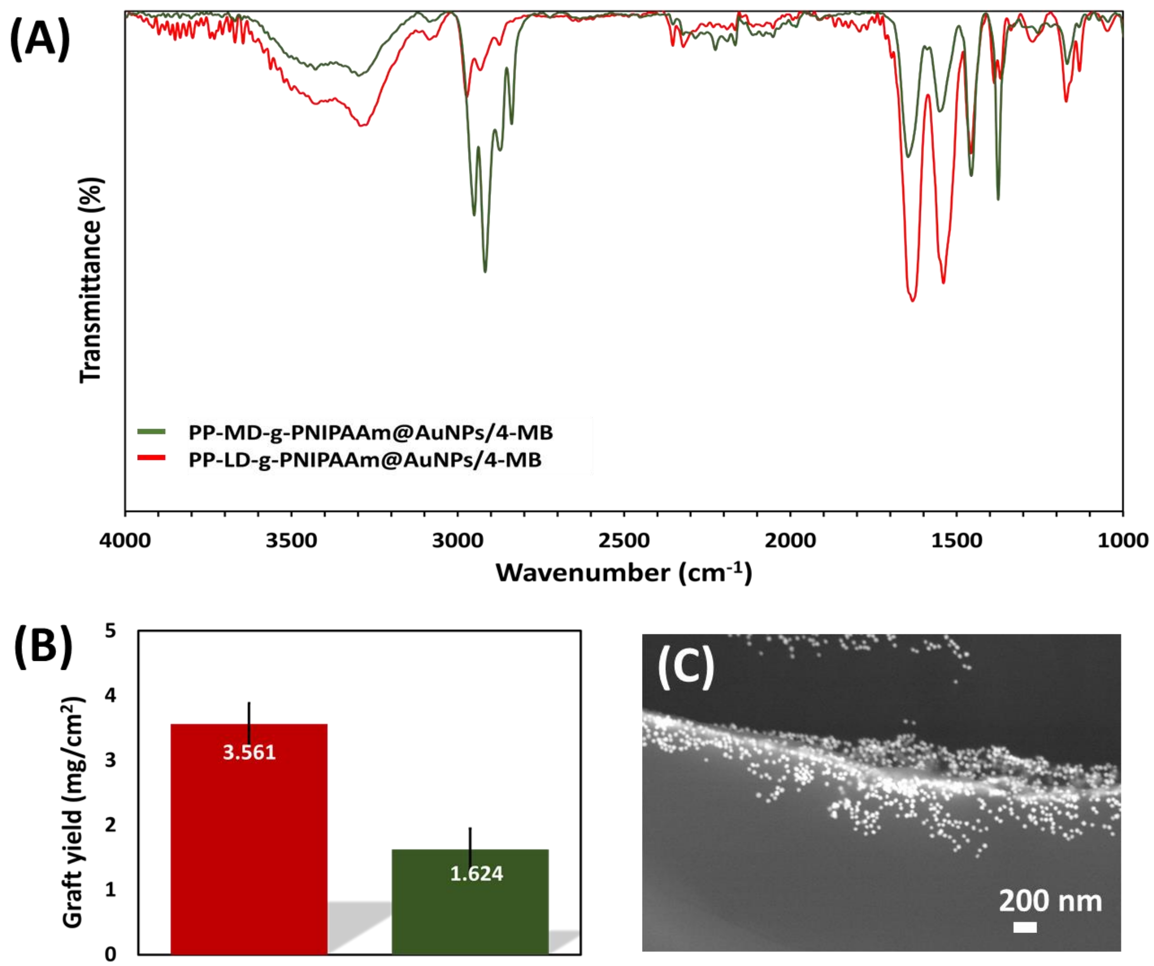

Figure S3. A) FTIR spectra of PP-LD-g-PNIPAAm@AuNPs/4-MB and PP-MD-g-PNIPAAm@AuNPs/4-MB with AuNPs average size of 60 nm. B) Graft yield values of both samples: LD (green bar) and MD (red bar). (C) SEM micrograph of AuNPs adhered to PP-MD fibre and under PNIPAAm layer. The darkness contrast is due to the use of backscattering detector, which is able to discriminate between the Au-spherical NPs (white color contrast) and the gel plus PP fibres (with grey color).

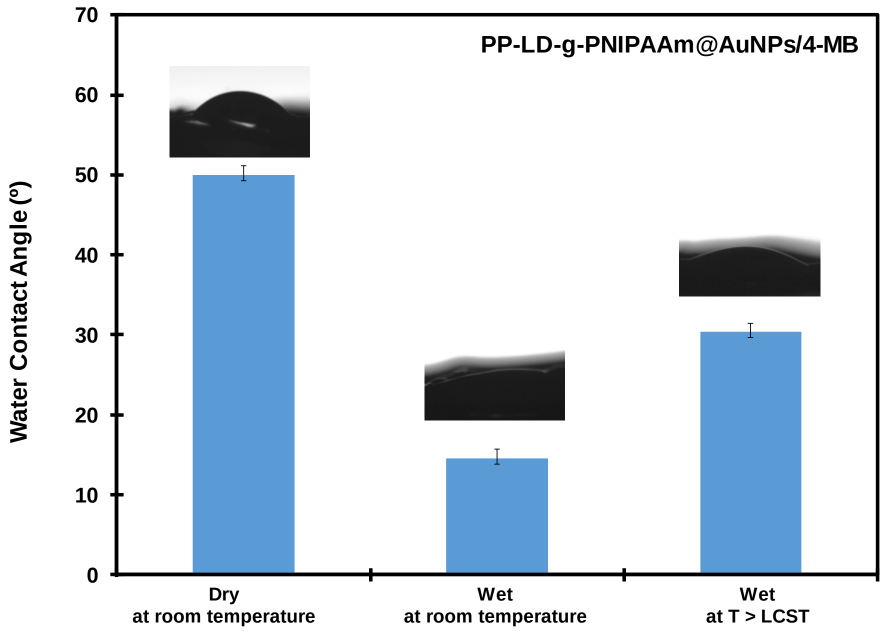

Figure S4. Contact angle measurements of the PP-LD-g-PNIPAAm@AuNPs/4-MB grafted mesh at different temperatures (dry and wet conditions).

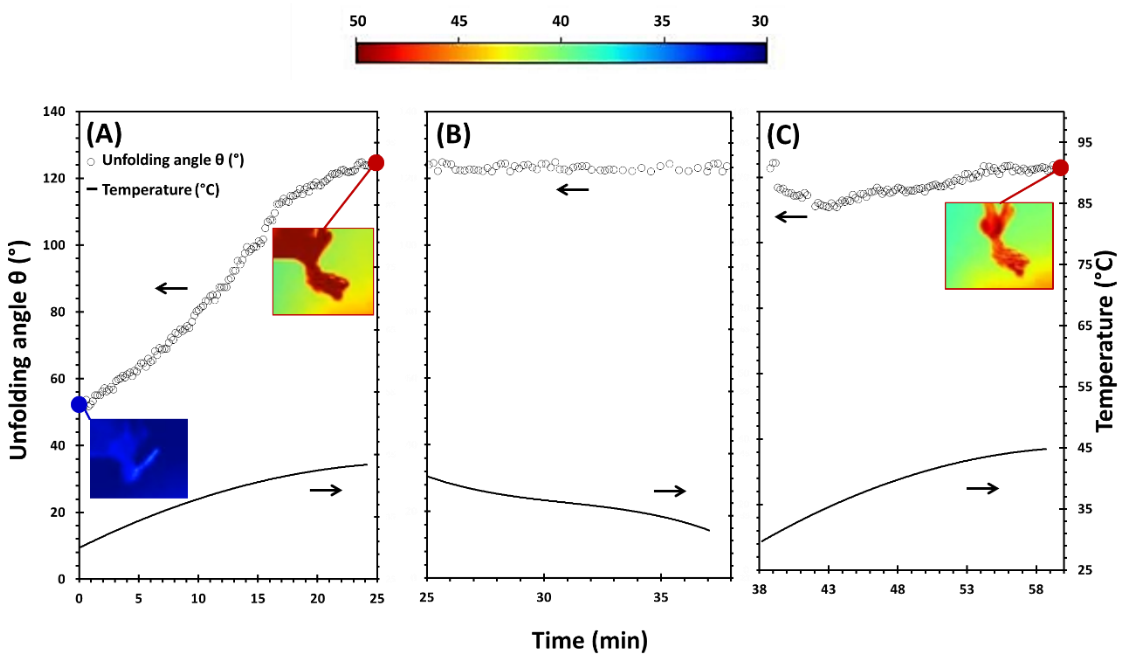

Figure S5. Variation of unfolding angles and temperature versus time for PP-LD@AuNPs/4-MB, *i.e.* sample without thermosensitive hydrogel, under cooling and heating successive experiments: (A) first heating, (B) first cooling and (C) second heating. The insets are infrared (IR) images of the modified mesh at the corresponding values of temperature and unfolding angles represented by blue and red dots. The color bar on top provides a key for the meaning of colors representing temperatures in IR images.
